# Cutaneous Groin Metastasis as a Rare Presentation of Advanced Urothelial Carcinoma of the Bladder

**DOI:** 10.7759/cureus.105163

**Published:** 2026-03-13

**Authors:** Kiran Ghotra, Miranda Garmo, Michaele Oostendorp

**Affiliations:** 1 Internal Medicine, Lake Erie College of Osteopathic Medicine, Erie, USA; 2 Internal Medicine, Corewell Health, Trenton, USA

**Keywords:** cutaneous metastasis, immune checkpoint inhibitors, lymphangitic spread, palliative care, urothelial carcinoma

## Abstract

Cutaneous metastases from urothelial carcinoma are rare and, when present, are associated with a poor prognosis. We report the case of a 70-year-old male with advanced urothelial carcinoma who developed biopsy-proven cutaneous metastases to the groin. The patient initially presented with hypoxia, worsening cancer-related pain, and a left groin rash. Before hospitalization, he had been treated with chemotherapy, radical cystectomy with ileal conduit, and pembrolizumab. Following discontinuation of immunotherapy because of immune-related toxicity, he later presented with hypoxia, worsening cancer-related pain, and a new groin rash that was ultimately confirmed to represent cutaneous metastasis. Imaging obtained during hospitalization revealed progression of metastatic disease involving the mediastinal lymph nodes, lungs, and bone. The patient ultimately died of disease progression. This case highlights the importance of recognizing atypical metastatic presentations, managing immunotherapy-related toxicities, and incorporating early palliative care discussions.

## Introduction

Muscle-invasive urothelial carcinoma is characterized by cancer that has spread into the muscle layer of the bladder wall. The bladder wall is composed of several layers, and most bladder cancers begin in the urothelium, also called the transitional epithelium, which is the inner lining of the bladder. Urothelial carcinoma, also known as transitional cell carcinoma, arises from the cells of the urothelium. Bladder cancer can progress and spread to nearby tissues. It is associated with a higher risk of metastasis, with the most common metastatic sites involving the lymph nodes, bone, lungs, liver, and peritoneum. Cutaneous involvement is uncommon and usually indicates widespread systemic disease [1-11]. Diagnosis is typically made with cystoscopy and transurethral resection of the bladder tumor [12]. This provides histological confirmation and helps determine the depth of invasion. Staging typically includes CT or MRI of the abdomen and pelvis, as well as chest imaging, to assess for metastatic disease [12].

For localized muscle-invasive urothelial carcinoma, definitive treatment includes neoadjuvant cisplatin-based chemotherapy followed by radical cystectomy with pelvic lymph node dissection [13]. In metastatic disease, systemic therapy is used, including platinum-based chemotherapy such as cisplatin [13]. Immune checkpoint inhibitors, including pembrolizumab, atezolizumab, nivolumab, durvalumab, and avelumab, have demonstrated efficacy in metastatic urothelial carcinoma but are associated with immune-related adverse events. The most frequent events include hypothyroidism, rash, pruritus, colitis, and pneumonitis. While these toxicities can necessitate treatment discontinuation, the development of immune-related adverse effects may paradoxically correlate with improved clinical outcomes.

## Case presentation

**Figure 1:**
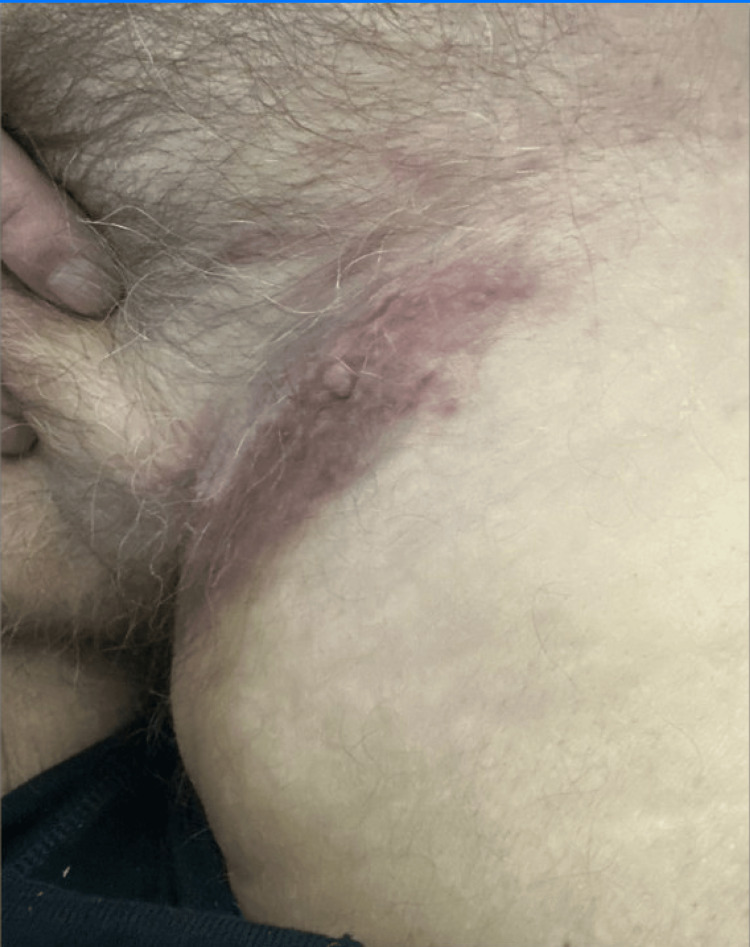
Patient’s groin showing cutaneous metastatic lesions one day after admission.

**Figure 2:**
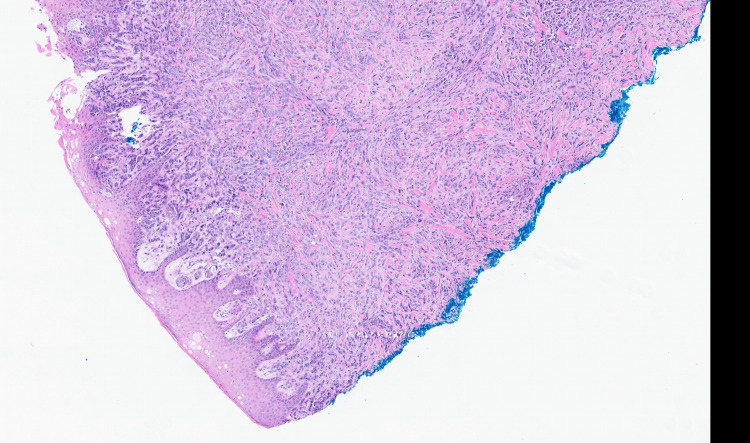
H&E-stained biopsy specimen from the groin rash demonstrating poorly differentiated carcinoma.

A 70-year-old male with muscle-invasive bladder cancer status post chemotherapy and radical cystectomy with ileal conduit, chronic obstructive pulmonary disease, hypertension, chronic kidney disease stage 3a, and prior pembrolizumab therapy presented to the hospital. He reported shortness of breath, hypoxia, worsening bone pain, and a painful left groin rash (Figure 1). Initial laboratory evaluation demonstrated leukocytosis, normocytic anemia, and renal dysfunction, with significantly elevated blood urea nitrogen and creatinine levels (Table 1). Computed tomography angiography was obtained because of worsening hypoxia despite medical therapy and supplemental oxygen, and revealed multiple sclerotic osseous lesions, bilateral pulmonary nodules, and mediastinal and intra-abdominal lymphadenopathy [1,2,4,5,7,9]. Further evaluation of the left groin rash included a punch biopsy, which showed poorly differentiated carcinoma with lymphangitic spread, consistent with metastatic urothelial carcinoma (Figure 2). The cutaneous lesions appeared as multiple subcutaneous nodules in the groin region. The patient was treated symptomatically with analgesics, which did not relieve his pain.

**Table 1:** Significant laboratory findings at admission.

Laboratory Test	Patient Value	Reference Range
WBC count (×10³/µL)	13.4	4.0-11.0
Hemoglobin (g/dL)	10.6	13.5-17.5
Blood urea nitrogen (mg/dL)	72	6-20
Creatinine (mg/dL)	1.75	0.8-1.3

**Figure 3:**
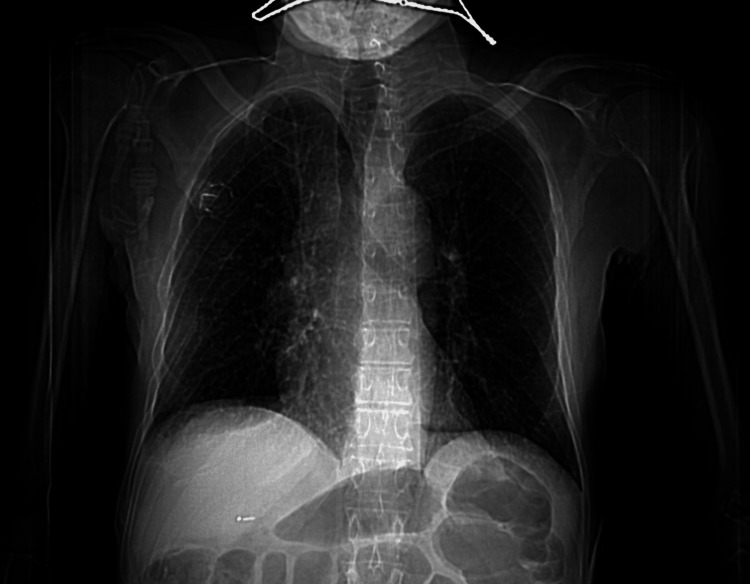
CT image demonstrating bulky mediastinal lymphadenopathy with mass effect on adjacent cardiac structures.

Due to the extent of disease spread, hospice and palliative care were consulted. The prognosis was poor, and care was transitioned to comfort-focused measures. The patient deteriorated significantly on hospital day 6 with severe hypoxia and cardiac compromise, most likely due to the mediastinal lymphadenopathy (Figure 3). Despite prolonged resuscitative efforts in accordance with Advanced Cardiovascular Life Support (ACLS) guidelines, the patient was pronounced deceased prior to discharge. The interval from diagnosis of cutaneous metastases to death was consistent with the poor prognosis typically associated with this presentation [1,3,10,11].

## Discussion

Cutaneous metastases from urothelial carcinoma represent a rare but ominous manifestation of advanced disease. Most reported cases involve the abdominal wall or extremities; groin involvement, as observed in this patient, is particularly uncommon. The histopathologic finding of lymphangitic spread in this case is consistent with aggressive disease biology and portends a particularly poor prognosis [3-5].

Studies have reported that cutaneous metastases most commonly appear following radical cystectomy and systemic therapy, typically within the first year after surgery [1,4]. The development of skin metastases in patients with urothelial carcinoma should prompt comprehensive restaging, as they frequently occur in the context of widespread systemic disease involving visceral organs [1,3,5,10,11].

The immune-related colitis experienced by this patient represents a well-recognized complication of checkpoint inhibitor therapy. Colitis occurs in approximately 2% to 8% of patients receiving single-agent PD-1/PD-L1 inhibitors and may require treatment discontinuation and systemic corticosteroid therapy. While immune-related adverse events can limit therapy duration, emerging evidence suggests that their development may correlate with improved treatment response and survival outcomes in urothelial carcinoma, with patients who experience immune-related adverse events demonstrating significantly improved overall and progression-free survival [8]. Despite these therapeutic advances, this patient’s disease progressed rapidly following pembrolizumab discontinuation. The constellation of mediastinal, pulmonary, and osseous metastases, in addition to cutaneous involvement, exemplifies the aggressive nature of advanced urothelial carcinoma.

This case underscores several important clinical considerations. Clinicians should remain highly vigilant for cutaneous metastases in patients with urothelial carcinoma who present with new skin lesions, and early biopsy should be pursued for definitive diagnosis. Management of immune-related adverse events requires careful balancing of toxicity mitigation against potential therapeutic benefit, recognizing that immune-related adverse events may function as biomarkers of treatment response. Given the typically limited survival associated with cutaneous metastases, early integration of palliative care is essential to optimize symptom management [1,5,8,11].

## Conclusions

Cutaneous metastasis from urothelial carcinoma is an uncommon but highly significant finding that typically reflects widespread, aggressive disease with a poor prognosis. This case highlights a rare presentation of groin cutaneous metastasis in muscle-invasive bladder cancer complicated by immune-related toxicity from checkpoint inhibitor therapy. Key clinical lessons include the importance of early recognition and biopsy of atypical skin lesions in patients with urothelial carcinoma, careful monitoring and management of immunotherapy-related adverse events, and timely incorporation of palliative care to optimize patient-centered outcomes in this challenging clinical scenario.
